# Spinal Cord Infarction in the Course of a Septic Shock: About One Case and Review of the Literature

**DOI:** 10.1155/2017/1571048

**Published:** 2017-02-20

**Authors:** P. Henin, A. Molderez, V. Huberlant, H. Trine

**Affiliations:** Groupe Jolimont, Centre Hospitalier de Jolimont, 159 rue Ferrer, 7100 Haine Saint Paul, Belgium

## Abstract

We report the case of a patient admitted to our intensive care unit in the course of a septic shock, secondary to cholangitis. After rapid hemodynamic stabilization, antibiotherapy, and endoscopic extraction of bile ducts stones, she appeared to have developed flaccid paraplegia. The suspected diagnosis of medullar ischemia was confirmed by typical MRI findings. This case stresses the potential pathogenic role of hypotension in medullar ischemia and the place of magnetic resonance imaging (MRI) as a reliable diagnostic tool.

## 1. Introduction

Medullar ischemia is a rare and severe condition [1] that can lead to death or persistent paraplegia [2–4].

Its most usual etiologies are thoracoabdominal aortic surgery, trauma, or cardiovascular diseases. It can also be due to deep hypotension and global ischemia [4–6].

We report a seldom case of medullar ischemia occurring in the course of a septic shock. We also describe the typical magnetic resonance imaging (MRI) findings, most accurate tool for diagnosis confirmation [1, 3, 4, 7–10].

## 2. Case Report

A 55-year-old Caucasian woman was admitted through the Emergency Room with severe abdominal pain and dehydration. Her main past medical history included diabetes, active alcoholism, smoking, chronic pancreatitis, and hypercholesterolemia. She had a recent episode of acute on chronic alcoholic pancreatitis, with transient acute renal failure. She was transferred to our intensive care unit in septic shock with severe hypotension (MAP < 40 mmHg at the time of admission) and transient sinus bradycardia.

She also had fever, clinical jaundice, and peripheral signs of hypoperfusion. The neurological exam showed impaired consciousness but no motor or sensitive deficit.

Laboratory data showed lactic metabolic acidosis, acute renal failure, and cholestasis. There was no coagulation disorder.

An abdominal CT scan showed hepatic steatosis, pancreatic calcifications, gallbladder hydrops and lithiasis, and enlargement of the extrahepatic bile ducts. Ultrasonography confirmed the suspected diagnosis of cholangitis: enlargement of the choledochus (12 mm), occluded in its distal part by a 9 mm lithiasis.

Within the first hour, the MAP was restored to normal range (>65 mmHg). She received empirical antibiotherapy (Amoxicillin-Clavulanate) and an urgent ERCP was performed, with successful extraction of the common bile duct stone.

On the next day, her clinical exam confirmed the recovery of stable hemodynamic parameters, with normal urine output. Lactic acidosis was corrected, and renal function and liver enzymes were improving.

Unfortunately, the patient appeared to have developed complete flaccid paraplegia. A thorough neurological exam showed a sensory (pain and temperature) and motor deficit at T10 level and loss of sphincter control. We suspected spinal cord ischemia.

Somatosensory evoked potentials were compatible with that diagnosis.

**Figure 1:**
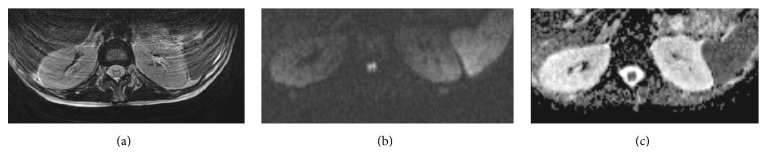
(a) The sagittal T2-weighted image shows a hyperintense lesion of the conus medullaris. (b) High signal is observed at the corresponding level on axial DWI. (c) Low ADC value on the ADC map confirmed the hypothesis of spinal cord infarction.

MRI demonstrated on the sagittal T2-weighted image a hyperintense lesion of the conus medullaris and high signal was observed at the corresponding level on axial DWI (Diffusion Weighted Imaging); low ADC (Apparent Diffusion Coefficient) value on the ADC map confirmed the hypothesis of spinal cord infarction (Figure 1).

The patient remained stable and further recovered from all her biological disorders. Bacteriology remained negative and antibiotics were discontinued on day 5.

Unfortunately, apart from discrete contraction of the proximal muscles of the right leg, the patient did not recover from her neurologic disorder and had persistent flaccid paraplegia, abolition of temperature and pain sensitivity, and sphincter dysfunction, despite thorough multidisciplinary rehabilitation.

She died a few months later in the rehabilitation center, in the course of an acute myocardial infarct.

## 3. Discussion

Acute onset of paraplegia is rarely seen in the ICU setting. The differential diagnosis will mainly be orientated by clinical context: recent history of trauma, lumbar or epidural puncture, recent aortic surgery, recent history of hemodynamic instability (shock or global hypoperfusion), cardiovascular comorbidities, especially with thromboembolic events, anticoagulant therapies, systemic inflammatory diseases, or cancers.

We can distinguish extramedullar and medullar etiologies.

Extramedullar etiologies include polyradiculitis (Guillain-Barre syndrome) [11] or can be part of the clinical manifestations of ion disorders [12] like hyperkaliemia or hypercalcemia. Their onset will usually be more subacute. Psychogenic disorders can also mimic paraplegia [13].

Medullar etiologies are more relevant to our discussion. They can be traumatic: vertebral trauma with cord lesion [14], through mechanisms of transection, compression, contusion, or vascular compromise, and spinal epidural bleeding after puncture [15].

Medullar etiologies can also be nontraumatic: medullar ischemia or thromboembolism, as discussed in this case report, spinal bleeding, spontaneous [16] or under anticoagulant therapy [17], tumor, or abscess compression [18, 19].

Other nontraumatic medullar etiologies of paraplegia can be seen, but their course is usually more subacute, not as sudden as in our case, and will often be part of a more complex clinical situation: hepatic myelopathy in liver cirrhosis [20], paraneoplastic necrotizing myelopathy [21], systemic inflammatory diseases like multiple sclerosis or sarcoidosis [22–24], or infectious diseases like viral or bacterial myelitis (e.g., Lyme's disease or CMV). Some (around 15%) cases of transverse myelitis remain unclear and are referred to as idiopathic [25].

Medullar ischemia is a rare diagnosis [1]. Its incidence is difficult to evaluate and few series are published [1–7, 9, 10, 26–28]. Anterior spinal artery syndrome (ASAS) is the most usual clinical presentation [1]. This syndrome results from an infarction of the anterior two-thirds of the spinal cord. The midthoracic level is usually seen as the watershed zone for ischemic vulnerability, but some authors argue that lower (lumbar or lumbosacral) levels seem most vulnerable to global ischemic events [4, 29].

The common symptoms are sudden weakness under the level of ischemia, flaccid paraplegia, areflexia, urinary bladder and anal sphincter dysfunction, and loss of pain and temperature perception without proprioceptive disorder (no involvement of the posterior third of the spinal cord) [4]. The onset is usually sudden, with maximal impairment often reached within the first hour [10]. Severity of impairment can be defined using the American Spinal Injury Association (ASIA) scoring [8] as follows:

1. Complete: no motor or sensory function is found in the lowest sacral segment (S4-S5).
2. Incomplete: sensory function is found below neurologic level and in S4-S5; no motor function is found below neurologic level.
3. Incomplete: motor function is preserved below neurologic level and more than half of the key muscle groups below neurologic level have a muscle grade less than 3.
4. Incomplete: motor function is preserved below neurologic level and at least half of the key muscle groups below neurologic level have a muscle grade greater than 3.
5. Normal: sensory and motor function is normal.

Initial impairment can be mild (ASIA scoring C or D), in about 40 to 50% of all cases, moderate (ASIA B), or severe (ASIA A), each around 25% [5, 10].

Etiologies of ASAS are multiple. The most common causes are surgery of the thoracoabdominal aorta, trauma, all vascular disorders like thromboembolism and inflammatory diseases, and global hypoxic-ischemic events such as cardiac arrest or severe hypotension [4, 9, 10]. Cardiovascular risk factors such as hypertension are common among patients with ASAS [2, 5, 7].

The best diagnostic tool for ASAS is neuroimaging with MRI. The typical findings are focal cord swelling with increased T2 signal in the central part of the spinal cord and in DWI (Diffusion Weighted Imaging), an increased medullar signal associated with a low ADC (Apparent Diffusion Coefficient) value [6, 10–12, 30].

The prognosis of ASAS is variable and can range from full recovery to long term persistence of complete paraplegia, loss of sensitivity, sphincter dysfunction, and chronic pain. The percentage of different outcomes is difficult to evaluate, depending on the author and because of the relatively small size of published series [1–7, 9, 10, 26–28, 31]. Death occurs in less than 10% [1, 5, 6] to more than 20% [9] of cases. Complete or satisfactory (ambulatory) recovery seems to occur in 25% of the survivors according to the pessimistic [9] and up to more than 50% according to the optimistic authors [1, 2]. Complete persistent impairment (wheelchair dependence, bladder catheter, loss of sensitivity, and chronic pain) also ranges from 20 [5] to more than 50% [9], depending on authors. The remaining patients will have partial recovery and a mix of motor, sensory, and autonomic sequelae.

Recovery occurs mainly in the first 2 to 4 weeks [4], but meaningful improvement can occur after several months in a significant minority of cases [10].

All authors agree that severity of the initial impairment is associated with a poor outcome [2, 4, 6, 9, 10]. The correlation between age at onset and outcome is disputed. Some authors find a better prognosis in younger patients [10], while others describe a poorer prognosis below age of 55 years [2].

Severe hypotension is a recognized etiological factor of spinal cord ischemia [29, 32], and our case shows the potential deleterious consequences of sepsis related shock on medullar perfusion. It stresses the importance of quickly restoring a MAP above 65 mmHg according to the surviving sepsis campaign guidelines [33], especially in patients with cardiovascular risk factors, in order to prevent, among other complications, medullar ischemia.

There is no specific therapy for ASAS. Treatment in the acute phase is thus mainly supportive. Selective injection of Dexamethasone and Urokinase in the Adamkiewicz artery (arteria radicularis magna) has been proposed in a small group of 3 patients [28].

During thoracoabdominal aortic surgery, lumbar CSF drainage can be used to protect medullar perfusion. The rationale is to maintain a spinal perfusion pressure (MAP − intrathecal pressure) above 70 to 80 mmHg, by supporting hemodynamics and draining CSF to maintain intrathecal pressure below 10 mmHg [34].

Other techniques are proposed in order to reduce the incidence of perioperative medullar ischemia, such as perioperative somatosensory evoked potentials [35], induced hypothermia [36], intrathecal papaverine [37], or epidural cooling [38].

## 4. Conclusion

Spinal ischemia is a severe condition. Its most common presentation is ASAS. It is a well known complication of thoracoabdominal surgery but can also result from traumatic, cardiovascular, or systemic disorders and from severe hypotension and global ischemia.

Its onset is sudden, and its prognosis is variable, from recovery to persistent paraplegia or death.

A severe initial impairment is related to a poorer prognosis, but recovery can occur even in severe cases, and delayed recoveries are not exceptional.

This case report shows the potential consequences of septic shock on medullar perfusion and stresses the importance of rapidly restoring MAP, according to the surviving sepsis campaign.

It also shows the key diagnostic role of MRI and its typical findings in cases of ASAS.
